# Prevalence of Co-Infections in Primary Care Patients with Medically Attended Acute Respiratory Infection in the 2022/2023 Season

**DOI:** 10.3390/v16081289

**Published:** 2024-08-13

**Authors:** Maja Sočan, Katarina Prosenc, Maja Mrzel

**Affiliations:** 1 National Institute of Public Health, 1000 Ljubljana, Slovenia; maja.mrzel@nijz.si; 2 National Laboratory for Health, Environment and Food, 1000 Ljubljana, Slovenia; katarina.prosenc@nlzoh.si

**Keywords:** respiratory viruses, co-infections, acute respiratory infections, primary care, sentinel, influenza, SARS-CoV-2, RSV

## Abstract

In the post-pandemic period, an endemic circulation of respiratory viruses has been re-established. Respiratory viruses are co-circulating with SARS-CoV-2. We performed a retrospective analysis of co-infections in primary care patients with medically attended acute respiratory infections (MAARI) who consulted from week 40/2022 to week 39/2023 and were tested for a panel of respiratory viruses. Out of 2099 samples tested, 1260 (60.0%) were positive for one virus. In 340 samples, co-infection was detected: two viruses in 281 (13.4%), three viruses in 51 (2.4%), and four viruses in eight (0.4%) samples. Respiratory viruses co-infected the patients with MAARI at very different rates. The lowest rates of co-infections were confirmed for influenza B (13.8%) and influenza A (22.9%) and the highest for human bocaviruses (84.0%) and human parechoviruses (82.1%). Co-infections were detected in 28.2% of SARS-CoV-2 positive samples. SARS-CoV-2 has never been co-infected with influenza B virus, enterovirus or adenovirus, although the latter was found as a co-infecting virus with all other respiratory viruses tested. The rate of co-infections decreased significantly with increasing age (*p*-value 0.000), and no difference was found regarding gender (*p*-value 0.672). It is important to understand the epidemiology of respiratory co-infections for prevention and management decisions in patients with MAARI.

## 1. Introduction

Acute respiratory infections (ARI) are caused by various viruses with similar but slightly different epidemiological and clinical characteristics. In the pre-pandemic COVID-19 period, the influenza virus and respiratory syncytial virus were two major viral pathogens causing the most substantial burden in young children and elderly adults [1,2]. The contribution of other respiratory viruses to the burden of ARI was likewise substantial. Infection with human metapneumovirus, parainfluenza viruses, adenoviruses and human coronaviruses leads to higher pressure on health care systems with an increased number of consultations in primary care, hospitalizations and, in individuals at risk, to unfavorable outcomes of the infection [3]. Acute respiratory infections cause excess winter mortality (EWM) and are used to estimate the impact of winter infections on deaths. 

Infections with respiratory viruses can occur throughout the year. In the temperate climate zone, the incidence of ARI increases with a decrease in air temperature and increased humidity in autumn and rises further over winter [4]. The decrease is gradual, and the season ends in April to May. Global patterns of respiratory virus activity have shown temporal differences in the incidence of respiratory virus–influenza virus timing, and the peak of the flu season is commonly later and more variable compared to the offset and peak of the RSV season. Rhinoviral infections increase even earlier compared to RSV—the incidence of rhinoviral infections increases in September, declines moderately over the winter and grows again in the spring [5]. The seasonal onset, peak and offset of respiratory viruses vary to some extent, but in most of the seasons, their circulation at least partly overlaps. 

Non-pharmaceutical interventions (NPIs) for the COVID-19 pandemic limited the spread of SARS-CoV-2. At the same time, NPIs reduced the circulation of other respiratory viruses substantially [6,7,8,9]. In the 2020/2021 season, the global incidence of the influenza virus decreased by more than 90%, and some other respiratory viruses were also almost undetectable, e.g., RSV and hMPV [6,7]. Interestingly, the circulation of rhinoviruses was preserved the most [10]. The decline in the circulation of respiratory viruses during the COVID-19 pandemic could also be at least partially explained by interference. An infection with two or more respiratory viruses can occur simultaneously or sequentially. Infection by a first virus could enhance or reduce infection and replication of a second virus, resulting in synergistic or antagonistic interaction [11,12]. Interference is regulated by interferons whose gene expression is regulated by non-coding RNAs (ncRNAs) coded into the pathogens’ genetic material [13]. The NPIs were gradually dropped, and social interactions normalized, which led to an increase in the incidence of ARI with changed epidemiological patterns. An unprecedented and unexpected summer surge in RSV activity occurred in 2021 [14,15]. The influenza seasonal pattern showed an exceptionally late onset, crossing the epidemic threshold in February 2022, with an overall shorter duration compared to every season since 2009 [16]. 

The overlapping seasonality of respiratory viruses raises the possibility of simultaneous infection with two or more respiratory viruses at the same time. Studies from the pre-pandemic period found various degrees of prevalence of co-infections and their impact on clinical course depending on the age group studied, timing of the study and number of respiratory viruses tested in one sample. Most studies confirmed a correlation between age and proportion of co-infections, with very young children having the highest prevalence of co-infections [17,18]. Pandemic studies assessing the respiratory viral interaction between SARS-CoV-2 and other viruses revealed that co-infection rates varied according to the country and period of the study [19].

The integration of COVID-19 in a sentinel surveillance system for influenza-like illness (ILI) and ARI was recommended by the WHO in late 2020 [20]. In Slovenia, testing for SARS-CoV-2 infection was integrated into the panel of respiratory viruses, which gave us the opportunity to study co-infections in medically attended acute respiratory infections (MAARI) for multiple viruses. 

In the present paper, we describe the rate and demographics of co-infections in MAARI patients in the 2022/2023 season detected through an integrated sentinel surveillance network in Slovenia.

## 2. Materials and Methods

### 2.1. Study Population 

The samples were obtained from patients who consulted for MAARI from 3 October 2022 (week 40/2022) to 1 October 2023 (week 39/2023) in outpatient clinics that take part in the national sentinel surveillance network for integrated surveillance of acute respiratory infections. The sentinel system provides seasonal, weekly, and year-round consultations for ILI and ARI.

The national sentinel surveillance network was established in 1999 and covers 4–5% of the Slovenian population. The network is nationwide and geographically representative. Patients from all age groups are included, with a slight overrepresentation of schoolchildren and an underrepresentation of elderly adults. 

In the beginning, the sentinel surveillance system was designed to detect the community transmission of influenza. Two indicators were used to identify the influenza season—weekly numbers of ILI (epidemiological indicator) and weekly numbers of positive samples for influenza virus from patients with signs and symptoms compatible with flu. The network developed over time, and the panel of respiratory viruses tested and detected in each sample gradually expanded. Testing for SARS-CoV-2 was added in the 2020/2021 season, and the sentinel surveillance was upgraded to integrated sentinel surveillance for ARI. Following the WHO recommendations, not only ILI but also ARI patients are now swabbed (nasal and throat swabs) and tested for respiratory viruses [20].

### 2.2. Sample Collection and Analysis

According to the protocol, the physicians participating in the sentinel network identify an appropriate case (fitting the definition of ARI) to collect nasal and pharyngeal swabs. The definition of ARI includes symptoms and signs of ARI infection, including coryza, sore throat, cough or shortness of breath, with or without fever, and a clinical assessment confirming the symptoms are a consequence of acute infection. It was recommended to only take samples from patients within the first five days of illness and to take samples from two patients per week. Swabs were stored and transported in 1.5 mL of viral transport medium. A short questionnaire was completed and sent together with the nasal/throat samples to the National Influenza Centre (NIC) National Laboratory of Health, Environment and Food. Nucleic acids were extracted and tested with real-time PCR, using AusDiagnostics Respiratory Viruses 16-well panel (product ref. number 20602) reagents, Mascot, New South Wales, Australia. This method detects influenza A (differentiating influenza types H1pdm09 and H3), B, RSV (differentiating RSV types A and B), adenoviruses, enteroviruses, rhinoviruses (RV), human bocavirus (hBoV), human metapneumovirus (hMPV, differentiating types A and B), parainfluenza viruses (differentiating all four types), human coronaviruses (hCoV), human parechovirus (hPeV), and SARS-CoV-2. Clinical performance of the method yields a sensitivity of 75% for hCoV, 82.9% for hBoV, 87.5% for parainfluenza viruses, 88.9% for hPEV, 91.7% for enteroviruses, and 100% for other targets. The specificity is 99.2% for hPeV, 99.7% for enteroviruses, and 100% for other targets (AusDiagnostics Instructions to use Respiratory Viruses (16-well) panel of assays). The extraction and internal PCR controls were included in each specimen; the positive (for all targets) and negative controls were used in each PCR run.

### 2.3. Statistical Analysis

We performed a descriptive analysis on 2099 samples. The combinations of co-infections with respiratory viruses were evaluated with a Pearson correlation coefficient. We compared mono- and co-infections between gender and age groups with the Chi-square test. Data were analyzed with IBM SPSS, version 27 (IBM Corp., Armonk, NY, USA).

## 3. Results

In the 2022/2023 season, 2099 patients (1136 females, 54.1% and 963 males, 45.9%) with ARI were tested for the panel of respiratory viruses within the national integrated sentinel surveillance for respiratory viruses in primary care. The samples were collected from all geographical areas of Slovenia. The median age of patients tested was 19, ranging from ≤1 to 103. The number of samples taken per age group was: 0–3 years—357 samples (17.0%), 4–7 years—247 samples (11.8%), 8–14 years—316 samples (15.1%), 15–19 years—139 samples (6.6%), 20–64 years—859 samples (40.9%) and ≥65 years—181 samples (8.6%).

Overall, 499 (23.8%) patients tested negative for all respiratory viruses included in the panel, 1260 (60.0%) patients were positive for one virus, 281 (13.4%) for two viruses, 51 (2.4%) for three viruses and eight patients (0.4%) were positive for four viruses. Altogether, there were 2007 respiratory viruses detected in the 2099 patients swabbed.

The number of detections per respiratory virus is presented in Table 1. 

**Figure 1:**
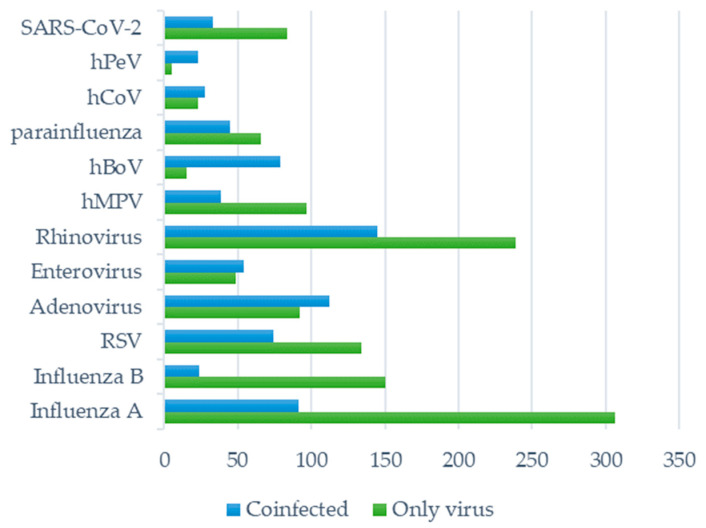
The number of ARI cases infected with one respiratory virus or in combination with other respiratory viruses.

Influenza viruses (A and B) were most frequently detected in nasal/pharyngeal swabs of ARI patients, followed by rhinoviruses, RSV, and adenoviruses. The detection rate of enteroviruses, hMPV, hBoV, parainfluenza viruses, hCoV, hPeV, and, interestingly, SARS-CoV-2, was less than 10%. The most rarely detected was hPeV. In Figure 1, the numbers of cases infected with a single respiratory pathogen or in combination with other viruses are presented. 

Three hundred and seventy-two influenza viruses were subtyped; 182 A(H1pdm09) and 190 influenza A(H3N2) cases were confirmed. Among 208 RSV-confirmed infections, there were 18 RSV A and 190 RSV B infections. There were 111 infections with parainfluenza viruses, and type 1, 2, 3, or 4 was found in 35, 5, 39, and 32 MAARI patients, respectively. 

**Figure 2:**
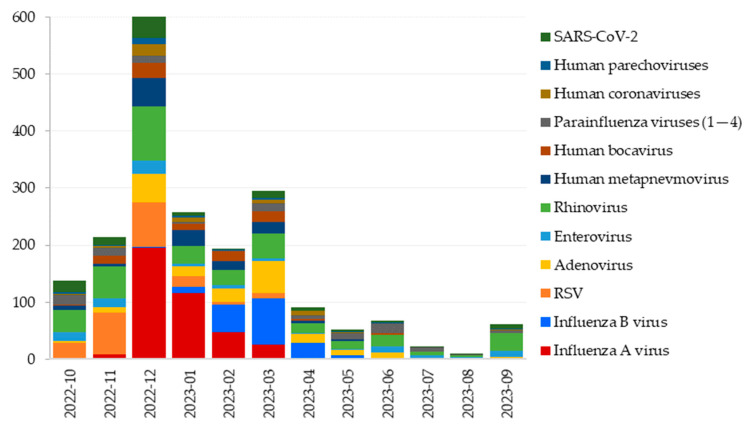
The number of monthly detections of respiratory viruses in the 2022/2023 season in Slovenia.

The occurrence of respiratory viruses by month of confirmation is shown in Figure 2. Some viruses (e.g., rhinoviruses, adenoviruses, parainfluenza viruses, and SARS-CoV-2) were detected in every month of the year, while the occurrence of others was limited mainly to the winter part of the season. 

Among those who tested positive for respiratory viruses, there were 340 patients whose samples contained the genome of at least two viruses, i.e., were co-infected. Correlations between all observed combinations were very low (Supplementary Materials, Tables S1 and S2). The combinations of co-infections with respiratory viruses are shown in Table 2.

Respiratory viruses co-infected the patients with MAARI at very different rates. Human bocavirus and human parechovirus were detected in co-infections with at least one additional virus in 84.0% and 82.1% of cases, respectively. The lowest rates of co-infections were confirmed for influenza B (13.8%) and influenza A (22.9%). Adenoviruses, enteroviruses, and hMPV were found in co-infections in approximately half of the cases (54.9%, 52.4%, and 54.9%, respectively). Parainfluenza viruses, rhinoviruses, RSV, hMPV, and SARS-CoV-2 were co-infected with at least one additional virus in 40.5%, 37.8%, 35.6%, 28.7%, and 28.2% of cases, respectively.

We studied the impact of gender and age on co-infections with respiratory viruses. As shown in Table 3, there were no statistically significant differences regarding gender when comparing the number of mono-infections to co-infections. The rate of co-infections decreased significantly with increasing age.

## 4. Discussion

The COVID-19 pandemic began in the first months of 2020. The WHO declared a Public Health Emergency of International Concern (PHEIC) on 30 January 2020. After more than three years of the pandemic and several waves of different severity, the WHO announced that the pandemic was not over, but from 12 June 2023, COVID-19 was no longer a PHEIC. However, the public health measures that aimed to counteract the rapid spread of SARS-CoV-2 were relaxed in most countries before the WHO declaration. Schools and kindergartens reopened, public mass gatherings such as sports events, concerts and so on were allowed, and a nearly normal respiratory season was expected for 2022/2023 [21]. With the relaxation of the NPIs, concern was expressed that a “rebound phenomenon” would occur in the coming seasons of respiratory infections. Not only would an increase in the intensity of the circulation of respiratory viruses outside the expected time frames occur, but a significantly increased number of patients with MAARI and severe acute respiratory infections (SARI) would also over-burden healthcare systems [22,23,24,25]. 

In the present study, we confirmed a high proportion of patients with MAARI (76.2%) who had a positive PCR test for at least one of the respiratory viruses in the upper respiratory tract swabs. Influenza A and rhinoviruses were most often confirmed. The high frequency of confirmed influenza cases is not unexpected, as during the pre-pandemic period, participating doctors took swabs mainly from patients with suspected influenza. It might be that the number of influenza cases in this sample was overestimated, which could be one of the limitations of the study. The 2022/2023 season was only the second season with a modified, integrated approach to swabbing, and not only patients with ILI but also ARI patients consulting with MAARI were swabbed. The frequency of rhinoviruses is likewise not surprising, as there are three types of rhinoviruses and more than 100 genotypes, without significant cross-immunity and with different genotypes circulating in the same season [5]. There are also more rhinoviral infections in young children, who in the present study contributed a significant proportion of the swabs. 

It is noteworthy that the proportion of co-infections in patients with MAARI was high. Approximately 16% of all MAARI patients included in the study, or 21.2% of all patients who tested positive for at least one respiratory virus, were co-infected. The abundance of various types and subtypes of viruses in specimens collected from patients within the sentinel network closely matched the abundance observed in all specimens tested nationwide. For example, 70% of the influenza-positive specimens in the sentinel network were influenza A, compared to 79% in all specimens tested in Slovenia during the 2022/23 season. Within the influenza A in the sentinel network, the subtypes A(H1N1)pdm09 and A(H3N2) comprised 49% and 51%, respectively, while in all specimens, these subtypes were 52% and 48%, respectively. Similarly, the subtypes of RSV were well-aligned: 9% of RSV A and 91% of RSV B in the sentinel network, compared to 11% of RSV A and 89% of RSV B in all specimens. This consistency in subtype distribution indicates a good representativeness of sentinel sampling in this regard.

In the first pandemic winter season, there was a concern that the extent of the simultaneous circulation of influenza and SARS-CoV-2 would put an additional burden on healthcare systems. In particular, it was pointed out that co-infections of these two viruses might lead to a very unfavorable disease outcome. Studies investigating co-infections in 2020, 2021 and the first half of 2022 confirmed a low percentage of patients co-infected with influenza and SARS-CoV-2 or other respiratory viruses [19,26,27,28]. During the pandemic period with strict NPIs in force, the incidence of respiratory viruses, including influenza, was low, and thus, the probability of co-infections was low as well. A systematic review of 95 studies revealed that the pooled prevalence of SARS-CoV-2 co-infections with influenza virus was 2.45% [23]. An earlier systemic review, including studies until May 2020, found an even lower overall proportion (0.7%) of influenza and SARS-CoV-2 co-infections [19]. Both studies confirmed more co-infections in critically ill patients compared to mono-infected patients. During the first pandemic season of 2020/2021 in Slovenia, significantly fewer samples from MAARI patients were collected in the national sentinel system, and co-infections were less common (10%), as NPIs were strictly implemented, including the closure of schools and kindergartens. In the second pandemic year the proportion of co-infections increased to 15%, with fewer quadruple infections compared to the 2022/2023 season. In the 2022/2023 season, SARS-CoV-2 infected patients were concurrently infected with influenza A virus, rhinovirus, or RSV virus in 9.4%, 7.7%, and 6.8% of cases, respectively. Interestingly, SARS-CoV-2 was never co-infected with influenza B virus, enterovirus, or adenovirus, although the latter was found to be a co-infecting virus with all other respiratory viruses tested.

Furthermore, the most frequent concurrent infection was with rhinoviruses, as 23.5% of PCR-positive samples for adenoviruses were simultaneously positive for rhinoviruses. Both viruses (rhinoviruses and adenoviruses) co-circulate during the whole year, which gives a constant opportunity for co-infections. SARS-CoV-2 was circulating in the 2022/2023 season in relatively high numbers, as recorded by the national sentinel surveillance system, and yet there were no co-infections with adenoviruses. Swets et al. studied the outcomes of patients hospitalized with SARS-CoV-2 and co-infected with influenza virus, RSV or adenoviruses and found that the lowest number of patients were co-infected with adenovirus (3.5%) [29]. The varied incidence of co-infections with respiratory viruses supports the findings of studies on positive or negative interactions between viruses during co-infection. The course of infection of one virus might be influenced by prior or concurrent viral infection. Recent viral infection might cause a lower susceptibility to become infected by another respiratory virus [13]. Viral interference, i.e., the interaction of two respiratory viruses, might imply that cross-reactive immunity against a first virus prevents infection with a second virus, or a prior viral infection inducts a nonspecific innate immune response that prevents replication of a second virus [11,12].

Most of the patients infected with hBoV or hPeV were co-infected with one of the other respiratory viruses. Human bocavirus was often combined with influenza virus, adenovirus, or rhinovirus, and least often with SARS-CoV-2. Co-infection with other viruses was commonly observed in HBoV in previous studies, which raises the question of whether HBoV is a major cause of respiratory disease [30]. Human bocavirus infection might persist longer and might exacerbate other viral infections [31]. Infection with hPeV was rarely confirmed (1.3%) and, in most cases, was confirmed as a co-infecting virus with another virus from the panel. Human parechoviruses can cause respiratory, gastrointestinal, and central nervous system disease [32,33].

The uneven number of respiratory swabs by age group is one of the limitations of the study. A quarter of the swabs were taken from preschool children, and older adults were under-represented and thus, the applicability of the results to the general population is questionable. Still, the study provides information about rarely studied co-infections in older adults in a non-hospital setting. A second limitation is that the sampling was uneven across the year. As expected, there were significantly more samples taken during the peak of the respiratory virus season, and it is likely that a more systematic sampling approach—with the same number of swabs taken each month—might have given different results. On the other hand, this approach reveals “real world data”, which is an added value of the present study. 

## 5. Conclusions

In the study, a high proportion of MAARI patients were co-infected with an additional virus. This can be partly attributed to the larger proportion of children included in the study, the extensive panel of viruses tested, and also the post-pandemic intensive circulation of respiratory viruses after the worldwide cessation of non-pharmaceutical interventions. 

## Figures and Tables

**Table 1:** The number (percentage) of viral detections with PCR in the national integrated sentinel surveillance in the 2022/2023 season in Slovenia.

Respiratory Viruses	No. of Positive Samples (%)	No. of Negative Samples (%)
Influenza A virus	397 (18.9%)	1702 (81.1%)
Influenza B virus	174 (8.3%)	1925 (91.7%)
RSV	208 (9.9%)	1891 (90.1%)
Adenovirus	204 (9.7%)	1895 (90.3%)
Enterovirus	103 (4.9%)	1996 (95.1%)
Rhinovirus	384 (18.3%)	1715 (81.7%)
Human metapneumovirus	136 (6.5%)	1963 (93.5%)
Human bocavirus	94 (4.5%)	2005 (95.5%)
Parainfluenza viruses (1–4)	111 (5.3%)	1988 (94.7%)
Human coronaviruses	51 (2.4%)	2048 (97.6%)
Human parechovirus	28 (1.3%)	2071 (98.7%)
SARS-CoV-2	117 (5.6%)	1982 (94.4%)

**Table 2:** Co-infections in patients with outpatient medically attended acute respiratory infection (MAARI) in the 2022/2023 season in Slovenia.

Respiratory Virus	Influenza A	Influenza B	RSV	Adenovirus	Enterovirus	Rhinovirus	hMPV	hBoV	Parainfluenza	hCoV	hPeV	SARS-CoV-2
Influenza A												
Influenza B	3											
RSV	14	1										
Adenovirus	11	7	10									
Enterovirus	8	0	14	20								
Rhinovirus	29	10	24	48	0							
hMPV	6	3	3	9	4	11						
hBoV	24	6	6	22	7	19	6					
Parainfluenza	2	1	2	12	10	17	2	6				
hCoV	4	0	3	7	3	7	2	5	1			
hPeV	3	0	1	7	4	6	3	8	1	1		
SARS-CoV-2	11	0	8	0	0	9	1	3	3	3	1	

**Table 3:** Comparison of gender and age groups in patients with one viral infection or co-infection in the 2022/2023 season in Slovenia (Sig.—significance, *p*-values from Chi-square tests).

	Mono-Infected (%)	Co-Infected (%)	All (%)	Sig.
Gender				0.672
Male	573 (78.3.5%)	159 (21.7%)	732 (100%)	
Female	687 (79.1%)	181 (20.9%)	868 (100%)	
Age groups (years)				0.000
0–3	172 (53.9%)	147 (46.1%)	319 (100%)	
4–7	158 (73.1%)	58 (26.9%)	216 (100%)	
8–14	186 (77.2%)	55 (22.8%)	241 (100%)	
15–19	84 (87.5%)	12 (12.5%)	96 (100%)	
20–64	543 (89.8%)	62 (10.2%)	605 (100%)	
≥65	117 (95.1%)	6 (4.9%)	123 (100%)	

## Data Availability

The data analyzed in this study is subjected to the following licenses/restrictions: The datasets are available in accordance with the national legislation. Requests to access these datasets should be directed to M.M., maja.mrzel@nijz.si.

## Supplementary Materials

The following supporting information can be downloaded at: https://www.mdpi.com/article/10.3390/v16081289/s1, Table S1. Co-infections in patients with outpatient medically attended acute respiratory infection (MAARI) in the 2022/2023 season in Slovenia with added *p*-values; Table S2. Co-infections in patients with outpatient medically attended acute respiratory infection (MAARI) in the 2022/2023 season in Slovenia with a complete correlation matrix.
