# Supplementary material for: Prevalence of Co-Infections in Primary Care Patients with Medically Attended Acute Respiratory Infection in the 2022/2023 Season
Source: Viruses. 2024 Aug 13;16(8):1289. doi: 10.3390/v16081289 (PMC11359868; doi:10.3390/v16081289)
Supplement: Supplementary file 1 [file viruses-16-01289-s001.zip › viruses-3122872-supplementary.pdf]

Supplementary information for:

Prevalence of Co-Infections in Primary Care Patients with Medically Attended Acute Respiratory Infection in the 2022/2023 Season

Maja Sočan[1]*, Katarina Prosenc[2] and Maja Mrzel[1]

[1] National Institute of Public Health, 1000 Ljubljana, Slovenia; maja.mrzel@nijz.si
[2] National Laboratory for Health, Food and Environment, 1000 Ljubljana, Slovenia; katarina.prosenc@nlzoh.si
* Correspondence: maja.socan@nijz.si

Table S1. Co-infections in patients with out-patient medically attended acute respiratory infection (MAARI) in the 2022/2023 season in Slovenia with added p-values.

Respiratory virus	Influenza A	Influenza B	RSV	Adenovirus	Enterovirus	Rhinovirus	hMPV	hBoV	Parainfluenza	hCoV	hPeV	SARS-CoV-2
Influenza A												
Influenza B	3***											
RSV	14***	1***										
Adenovirus	11***	7**	10*									
Enterovirus	8**	0**	14	20***								
Rhinovirus	29***	10***	24**	48*	0***							
hMPV	6***	3**	3**	9	4	11**						
hBoV	24	6	6	22***	7	19	6					
Parainfluenza	2***	1**	2**	12	10*	17	2*	6				
hCoV	4*	0*	3	7	3	7	2	5	1			
hPeV	3	0	1	7**	4*	6	3	8***	1	1		
SARS-CoV-2	11**	0**	8	0**	0*	9**	1*	3	3	3	1	

* p ≤ 0.05; ** p ≤ 0.01; *** p ≤ 0.001

Table S2. Co-infections in patients with out-patient medically attended acute respiratory infection (MAARI) in the 2022/2023 season in Slovenia with complete correlation matrix.

Respiratory virus		Influenza A	Influenza B	RSV	Adenovirus	Enterovirus	Rhinovirus	hMPV	hBoV	Parainfluenza	hCoV	hPeV	SARS-CoV-2
Influenza A	Corr.												
	Sig.												
	N												
Influenza B	Corr.	-0,13											
	Sig.	0,000											
	N	3											
RSV	Corr.	-0,10	-0,09										
	Sig.	0,000	0,000										
	N	14	1										
Adenovirus	Corr.	-0,11	-0,06	-0,05									
	Sig.	0,000	0,008	0,012									
	N	11	7	10									
Enterovirus	Corr.	-0,06	-0,07	0,03	0,07								
	Sig.	0,003	0,002	0,200	0,001								
	N	8	0	14	20								
Rhinovirus	Corr.	-0,14	-0,10	-0,06	0,04	-0,11							
	Sig.	0,000	0,000	0,008	0,042	0,000							
	N	29	10	24	48	0							
hMPV	Corr.	-0,10	-0,06	-0,07	-0,03	-0,02	-0,07						
	Sig.	0,000	0,008	0,002	0,207	0,273	0,001						

	N	6	3	3	9	4	11					
hBoV	Corr.	0,04	-0,01	-0,03	0,10	0,03	0,01	0,00				
	Sig.	0,094	0,493	0,242	0,000	0,244	0,623	0,969				
	N	24	6	6	22	7	19	6				
Parainfluenza	Corr.	-0,10	-0,06	-0,06	0,01	0,04	-0,02	-0,04	0,01			
	Sig.	0,000	0,004	0,003	0,690	0,040	0,404	0,040	0,628			
	N	2	1	2	12	10	17	2	6			
hCoV	Corr.	-0,04	-0,05	-0,02	0,02	0,01	-0,02	-0,02	0,04	-0,02		
	Sig.	0,041	0,030	0,330	0,328	0,744	0,393	0,453	0,063	0,283		
	N	4	0	3	7	3	7	2	5	1		
hPeV	Corr.	-0,02	-0,03	-0,02	0,06	0,05	0,01	0,02	0,14	-0,01	0,01	
	Sig.	0,265	0,109	0,259	0,006	0,021	0,666	0,360	0,000	0,683	0,693	
	N	3	0	1	7	4	6	3	8	1	1	
SARS-CoV-2	Corr.	-0,06	-0,07	-0,02	-0,08	-0,06	-0,07	-0,06	-0,02	-0,03	0,00	-0,01
	Sig.	0,007	0,001	0,253	0,000	0,011	0,002	0,011	0,303	0,176	0,923	0,642
	N	11	0	8	0	0	9	1	3	3	3	1

Corr.: Pearson correlation coefficient
